# Treatment of Acute Myeloid Leukemia in Elderly Patients With Azacitidine–Venetoclax Combination in Developing Countries: A Single-Center Experience

**DOI:** 10.7759/cureus.102308

**Published:** 2026-01-26

**Authors:** Rim Chakara, Aznag Mohamed Amine, Abderrahim Raissi

**Affiliations:** 1 Clinical Hematology, Mohammed VI University Hospital Center, Marrakesh, MAR; 2 Clinical Hematology, Avicenna Military Hospital, Marrakesh, MAR

**Keywords:** acute myeloid leukemia, azacitidine, elderly patients, middle-income country, toxicity, treatment outcomes, venetoclax

## Abstract

Background and objectives: Acute myeloid leukemia (AML) predominantly affects elderly patients and is associated with a poor prognosis. Therapeutic options remain limited for patients who are ineligible for intensive chemotherapy. Real-world data on the use of azacitidine-venetoclax (AZA-VEN) in North African populations are scarce. This study aimed to assess the effectiveness, composite complete remission rates, and safety profile of AZA-VEN in elderly AML patients treated at a Moroccan center.

Materials and methods: We conducted a retrospective study including patients aged ≥60 years who received AZA-VEN between July 2021 and August 2024 at the Military Hospital Ibn Sina in Marrakech. VEN was administered with a ramp-up protocol, combined with AZA and prophylactic voriconazole. Treatment response was assessed after the first cycle using bone marrow aspiration and blood counts. Overall survival (OS) was estimated using Kaplan-Meier curves.

Results: Eight patients were included, with a median age of 67 years and a male predominance. Seven patients had comorbidities. Four patients achieved complete remission (CR) after the first cycle, while two showed no response. Four patients experienced relapse or disease progression during follow-up. Grade 3-4 hematologic toxicities were frequent: neutropenia in all eight patients, anemia in seven patients, and thrombocytopenia in seven patients. All patients developed infectious complications. Median follow-up was 17 months, with a median survival of 25 months. Four out of eight patients were alive at two years.

Conclusions: Our single-center experience confirms that AZA-VEN is an effective and feasible therapeutic option for elderly AML patients, with a median survival of 25 months. Hematologic and infectious toxicities remain major challenges. In a North African context, limited access to VEN due to its cost can be partially mitigated through dose adjustments with azole antifungals. Further prospective multicenter studies are warranted.

## Introduction

Acute myeloid leukemia (AML) is a clonal hematologic malignancy of the bone marrow characterized by the uncontrolled proliferation of immature myeloid cells. It predominantly affects older adults and is marked by the accumulation of blast cells within the bone marrow, peripheral blood, and, in some cases, other tissues [1]. The median age at diagnosis is approximately 67 years, and the disease is associated with a poor prognosis, with a five-year survival rate below 32%, making it the most fatal among leukemic entities [2,3]. Older patients often present with multiple comorbidities, frailty, and reduced bone marrow reserve, limiting their eligibility for standard intensive chemotherapy [4].

Allogeneic hematopoietic stem cell transplantation remains the only potentially curative option; however, its use is restricted to a small subset of patients due to age, comorbidities, donor availability, and transplant-related risks [5]. Consequently, a significant proportion of elderly patients are ineligible for intensive therapy, highlighting the urgent need for alternative, less intensive but effective regimens [6,7].

In recent years, the combination of a hypomethylating agent such as azacitidine (AZA) with venetoclax (VEN), a selective BCL-2 inhibitor, has transformed the management of patients unfit for intensive chemotherapy. Clinical trials, including the pivotal VIALE-A study, have demonstrated substantial improvements in composite complete remission (CRc) rates, overall survival (OS), and progression-free survival compared with AZA alone [8,9]. These benefits are particularly pronounced in patients with adverse cytogenetic and molecular risk profiles [10,11].

However, real-world data remain limited, especially concerning treatment tolerability, severe neutropenia, infectious complications, including bacterial and fungal infections, and necessary dose adjustments, particularly when combined with azole antifungals [12]. Furthermore, data from low- and middle-income countries (LMICs) are scarce, and access to novel therapies such as VEN is often restricted due to cost and availability [13]. Single-center reports from LMICs provide valuable insights into treatment feasibility, clinical outcomes, and the practical management of elderly AML patients in resource-constrained settings [14].

In this context, the present study reports the experience of treating elderly AML patients at a Moroccan tertiary center with the AZA-VEN combination.

The primary objective of this study was to descriptively assess treatment effectiveness, including CR and OS, in a real-world cohort of elderly patients with AML treated with AZA-VEN.

The secondary objectives were to evaluate treatment tolerability and safety, with a particular focus on hematologic toxicity and infectious complications, and to assess the feasibility of implementing AZA-VEN therapy in a resource-limited setting where access to novel agents may be constrained.

This analysis provides important insights into real-world outcomes and practical challenges associated with the use of AZA-VEN in North Africa, a region where elderly AML patients face both clinical and logistical barriers to optimal care.

## Materials and methods

Study design and setting

We conducted a single-center retrospective study at the Department of Clinical Hematology, Ibn Sina Military Hospital, Marrakech, Morocco. The study aimed to evaluate the real-world effectiveness, safety, and feasibility of the AZA-VEN combination in elderly patients with acute myeloid leukemia (AML) who were ineligible for intensive chemotherapy. The study period extended from July 2021 to August 2024, and all data were collected and analyzed up to April 2025. The study was approved by the institutional ethics committee, and all patient records were de-identified to maintain confidentiality.

Study population

Patients were eligible for inclusion if they were aged 60 years or older, had a confirmed diagnosis of AML according to WHO criteria, and were considered ineligible for intensive chemotherapy due to advanced age, comorbidities, or frailty. Both treatment-naive and relapsed/refractory patients receiving at least one full cycle of AZA-VEN were included.

Exclusion criteria comprised: age under 60 years, incomplete medical records preventing assessment of treatment response or survival, or receipt of fewer than one full cycle of AZA-VEN. These criteria ensured the study population represented real-world elderly AML patients for whom AZA-VEN was indicated.

 Data collection

Demographic, clinical, laboratory, cytogenetic, molecular, and treatment-related data were collected retrospectively from hospital medical records. Baseline characteristics included age, sex, comorbidities, prior hematologic disorders (such as high-risk myelodysplastic syndrome or myeloproliferative neoplasms), and AML classification according to the 2022 European LeukemiaNet (ELN) risk stratification. Treatment-related variables included the number of AZA-VEN cycles, dose adjustments, and occurrence of toxicities or infections. All data were anonymized prior to analysis to comply with patient confidentiality requirements.

Missing data were handled using a complete-case approach. Variables with missing information were not imputed and were excluded from specific analyses when unavailable. The number of patients included in each analysis is specified where applicable.

Treatment protocol

AZA was administered at a dose of 75 mg/m² per day by subcutaneous or intravenous injection for five to seven consecutive days per 28-day cycle. VEN was initiated using a dose ramp-up schedule to reduce the risk of tumor lysis syndrome, starting at 100 mg on day 1, 200 mg on day 2, and 300 mg on day 3. From day 4 onward, VEN was continued at a daily dose of 100 mg due to concomitant administration of azole antifungal prophylaxis. Voriconazole was prescribed at a dose of 200 mg twice daily starting on day 4. All treatments were administered concurrently in 28-day cycles. Dose interruptions or delays were implemented in cases of prolonged cytopenias, infectious complications, or other treatment-related toxicities, in accordance with institutional guidelines.

Criteria for treatment interruption or delay included prolonged neutropenia or thrombocytopenia beyond day 28 without evidence of residual leukemia, grade ≥3 infectious complications, or other clinically significant toxicities. Supportive care measures were provided according to institutional practice and included antimicrobial prophylaxis, transfusion support, and growth factor use at the treating physician’s discretion.

Response and outcome assessment

Therapeutic response was evaluated after the first treatment cycle using bone marrow aspiration performed between days 28 and 42, in addition to peripheral blood count assessment. Response criteria were based on standard definitions, with CRc defined as complete hematologic remission (CR) or CR with incomplete hematologic recovery (CRi). Relapse or disease progression was documented during subsequent follow-up visits.

Statistical analysis

Descriptive statistics were used to summarize baseline characteristics, treatment responses, and adverse events. Continuous variables were reported as median and range, while categorical variables were presented as absolute numbers. OS was defined as the time from the initiation of AZA-VEN therapy to death from any cause or last follow-up and was estimated using the Kaplan-Meier method. Given the small sample size, percentages were avoided in reporting patient-level outcomes.

## Results

**Figure 1:**
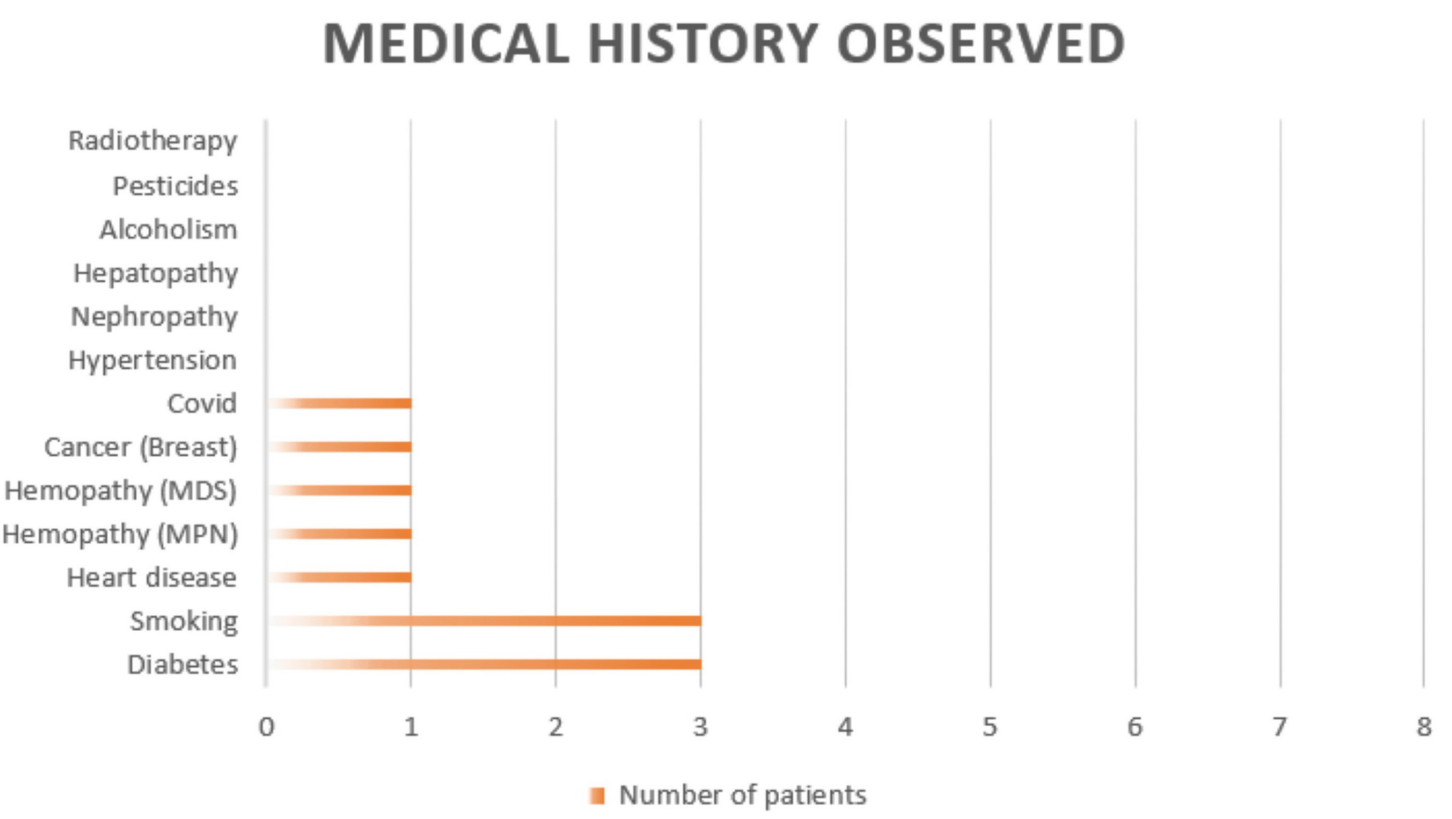
Medical history of the patients included in the study Image created by the authors with Microsoft Excel (Microsoft Corp., USA)

Eight patients were included in the analysis. The median age was 67 years, with a male predominance (six men and two women). Seven patients had at least one comorbidity (Figure 1).

One patient had high-risk myelodysplastic syndrome, one had a myeloproliferative neoplasm (myelofibrosis), and six patients had de novo acute myeloid leukemia. Four patients were relapsed or refractory following first-line therapy.

Clinical presentation was heterogeneous. Four patients presented with a tumor syndrome, all eight patients had anemia at diagnosis, and three patients presented with an infectious syndrome. No hemorrhagic manifestations were observed (Table 1).

**Table 1:** Clinical and baseline characteristics of the patients (n = 8)

Characteristic	Value
Median age, years (range)	67 (58–76)
Male sex	6
Female sex	2
ECOG performance status 0–1	3
ECOG performance status 2–4	5
Presence of comorbidities	7
Tumor syndrome at presentation	4
Anemic syndrome at presentation	8
Hemorrhagic syndrome at presentation	0

At diagnosis, all patients had anemia, while leukocytosis was observed in one patient and leukopenia in three patients. Neutropenia and thrombocytopenia were present in six and seven patients, respectively. The median peripheral blood blast percentage was 23 (range 0-53), and the median bone marrow blast percentage was 51 (range 14-88) (Table 2).

**Table 2:** Hematologic and biological findings at diagnosis (n = 8)

Parameter	Value
Anemia	8
Leukocytosis	1
Leukopenia	3
Neutropenia	6
Thrombocytopenia	7
Median peripheral blood blasts, % (range)	23 (0–53)
Median bone marrow blasts, % (range)	51 (14–88)
Laboratory evidence of disseminated intravascular coagulation	0

Cytogenetic analysis revealed a normal karyotype in four patients, while one patient each had a complex karyotype, trisomy 11 with diploidy, inversion 16, and translocation (8;12). Molecular testing was available for two patients and identified one IDH2 mutation and one ASXL1 mutation (Table 3).

**Table 3:** Cytogenetic and molecular characteristics

Characteristic	Number of patients
Normal karyotype	4
Complex karyotype	1
Trisomy 11 with diploidy	1
Inversion 16	1
Translocation (8;12)	1
IDH2 mutation detected	1
ASXL1 mutation detected	1

**Figure 2:**
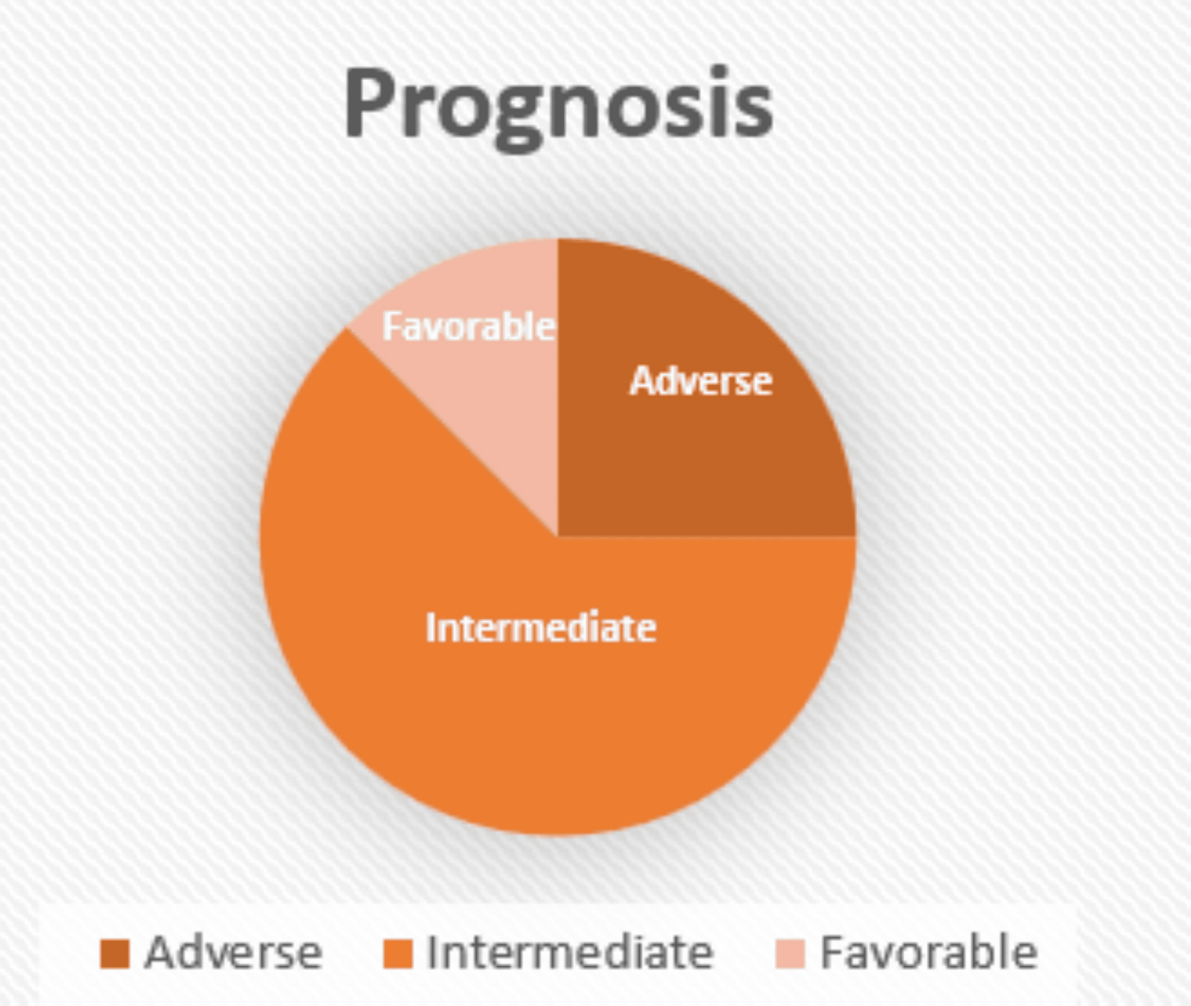
Prognostic classification according to European LeukemiaNet (ELN) 2022 Image created by the authors with Microsoft Excel (Microsoft Corp., USA)

Cytogenetic and molecular analyses allowed risk stratification according to the 2022 ELN classification [15], identifying one patient with favorable risk, five patients with intermediate risk, and two patients with adverse risk (Figure 2).

All patients received the azacitidine-venetoclax combination along with antifungal prophylaxis using voriconazole.

Two treatment subgroups were identified. Three patients received azacitidine-venetoclax as first-line therapy, while five patients received this combination as second-line therapy.

**Figure 3:**
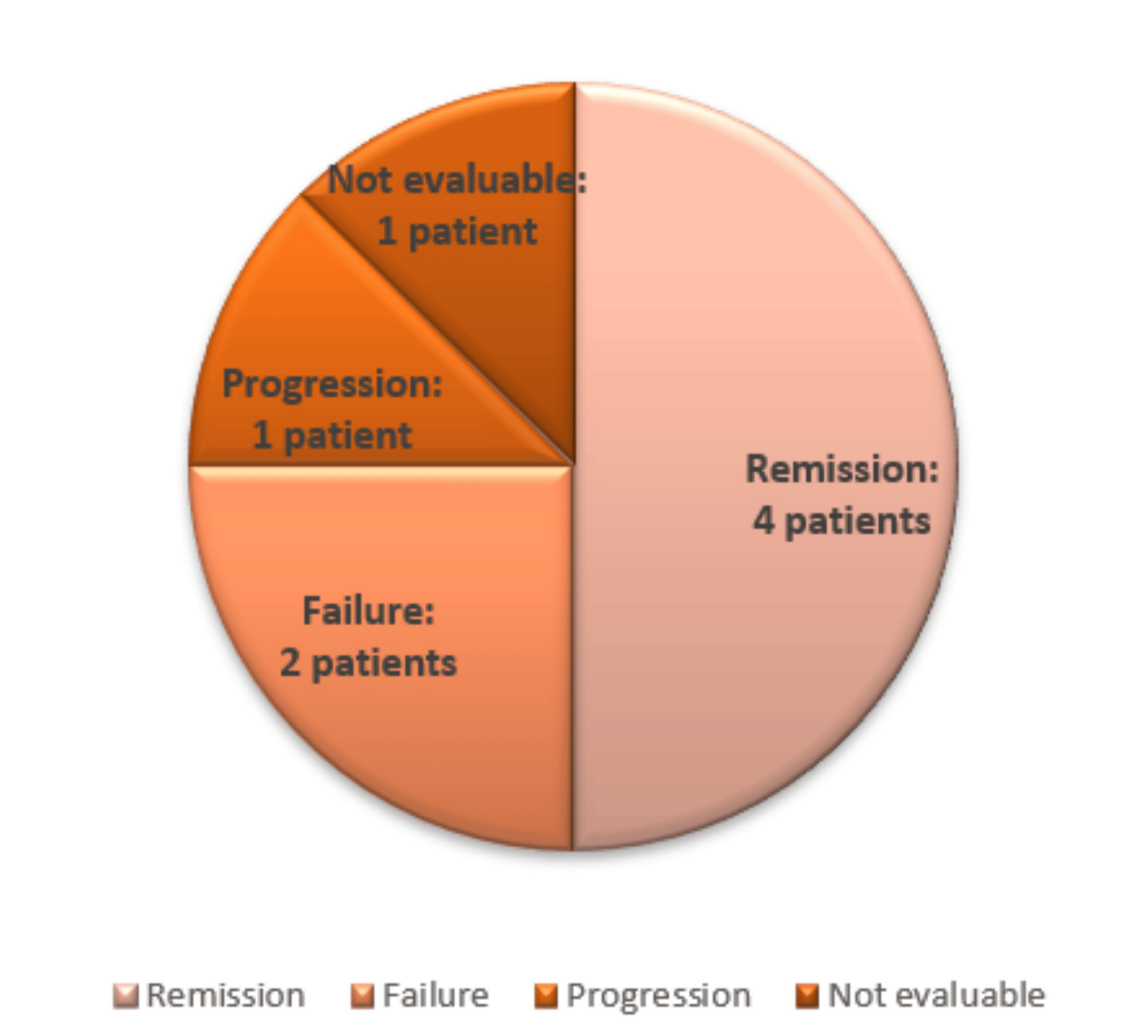
Outcomes of the assessment following the first treatment cycle Image created by the authors with Microsoft Excel (Microsoft Corp., USA)

A composite complete remission, defined as complete hematologic remission or complete remission with incomplete hematologic recovery, was achieved in four patients after the first treatment cycle. Two patients showed no response after the initial cycle. One patient experienced disease progression, and one patient could not be evaluated due to early mortality prior to response assessment (Figure 3).

During follow-up, four patients experienced relapse or disease progression while receiving therapy, with a mean of six treatment cycles administered. After a median follow-up of 17 months, four patients had died. Among these, two deaths were related to relapse or disease progression, one death occurred before treatment response assessment, and one occurred during the post-remission phase due to treatment-related toxicity.

Treatment-related toxicities were predominantly hematologic. Grade 3-4 neutropenia was observed in all eight patients. Grade 3-4 anemia and grade 3-4 thrombocytopenia were each observed in seven patients. Infectious complications occurred in all patients, most commonly presenting as febrile neutropenia.

**Figure 4:**
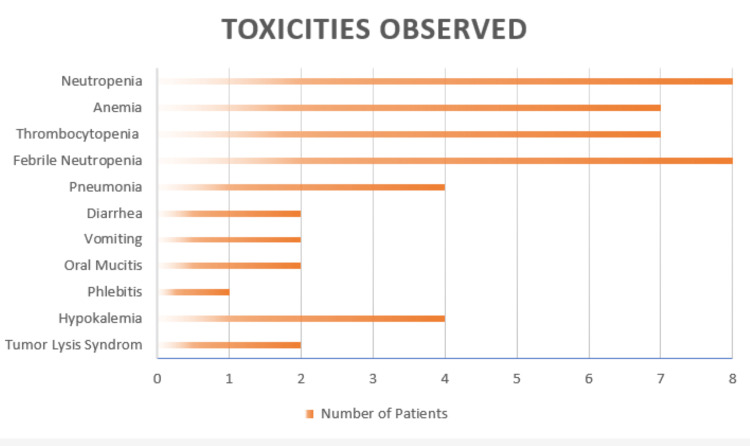
Toxicities observed with azacitidine plus venetoclax (AZA-VEN) Image created by the authors with Microsoft Excel (Microsoft Corp., USA)

Other complications included pneumonia in four patients, diarrhea in two patients, vomiting in two patients, oral mucositis in two patients, phlebitis in one patient, and hypokalemia in four patients. Biochemical tumor lysis syndrome was documented in two patients (Figure 4).

All eight patients developed febrile neutropenia during chemotherapy. A pathogen was identified in four cases. The isolated organisms included Klebsiella pneumoniae in one case, methicillin-resistant Staphylococcus epidermidis in one case, and Staphylococcus aureus in two cases. No pathogen was identified in the remaining four patients despite extensive microbiological investigations.

**Figure 5:**
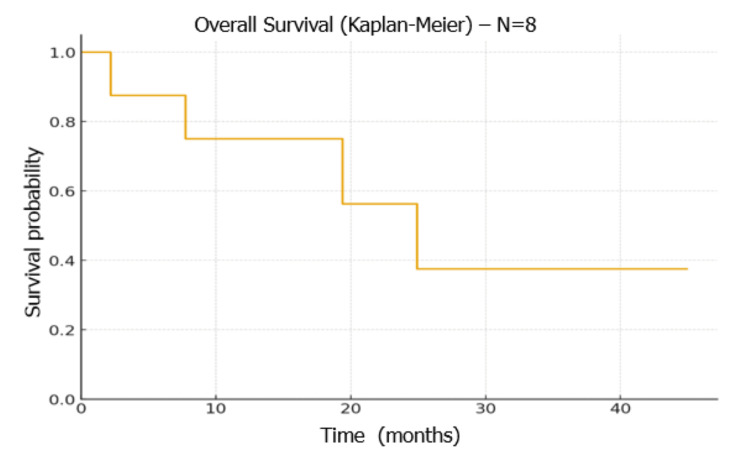
Overall survival Image created by the authors with the R statistical software (R Foundation for Statistical Computing, Vienna, Austria)

At the time of the last follow-up, four patients were alive. The median overall survival was 25 months (Figure 5).

## Discussion

The combination of AZA-VEN has markedly transformed the management of elderly patients with AML who are ineligible for intensive chemotherapy. In the landmark VIALE-A trial, the median OS was 14.7 months with AZA-VEN compared with 9.6 months with AZA alone, and CR rates were significantly higher in the combination arm (36.7% vs. 17.9%) [8,9]. These benefits were sustained beyond three years of follow-up, establishing the combined regimen as a new standard of care and highlighting the pivotal role of BCL-2 inhibition in regulating leukemic cell apoptosis [10,11].

Real-world analyses generally confirm the effectiveness of AZA-VEN, while highlighting variability according to clinical setting, resource availability, and patient population. A retrospective study by Yu et al. (2024) in China, including 57 elderly patients (mean age 70.0 years), reported an overall response rate (ORR) of 87.5%, a CR rate of 68.8%, and measurable residual disease (MRD) negativity in 58.3% of patients [16]. Similarly, a cohort of 587 patients treated with AZA-VEN in the UK National Health Service achieved a CR/CRi rate of 67%, with 30- and 60-day mortality rates of 5% and 8%, respectively, and a median OS of 13.6 months [17]. These findings suggest that AZA-VEN provides a clear therapeutic advantage over AZA alone, although outcomes remain influenced by patient frailty, comorbidities, and quality of supportive care [12,13].

Prospective data from the national REVIVE study offer further insights into the real-world effectiveness of this regimen [18]. The initial phase included 70 elderly patients (median age 75.0 years), reporting a CR/CRi rate of 52.3%, with 23% of responders eligible for allogeneic stem cell transplantation [18]. An extension cohort conducted in Israel (127 patients) demonstrated a CRc rate of 57% and a median OS of 9.6 months, emphasizing the strong association between early treatment response and OS [19]. More recently, an abstract presented at the EHA 2024 congress reported a median OS of 26.9 months in patients treated with AZA-VEN, with even more favorable outcomes (31.0 months) in those achieving remission within the first month of therapy [20]. These variations reflect the influence of patient selection, follow-up quality, and management of treatment-related complications on outcomes.

Beyond high-income countries, studies in resource-limited settings have also reported promising results. In Brazil, a multicenter study including 114 patients with AML-predominantly high-risk-reported a CRc rate of 50% after the first treatment cycle, with frontline patients achieving 58% and relapsed/refractory patients 38%; early mortality was low at 8% [21]. In India, a cohort of 108 patients reported an ORR of 67% in the first-line setting, surpassing that achieved with intensive chemotherapy (47.8%), with two-year OS of 64.2% in frontline and 45.8% in relapsed/refractory patients [14]. Additional real-world experience from Thailand confirms the feasibility of AZA-VEN in lower-resource settings, reporting frequent toxicities and infections despite efficacy [22]. These findings confirm the feasibility and clinical benefit of AZA-VEN even in LMICs, provided hematologic and infectious toxicities are closely monitored [12,13,22].

In our single-center cohort in Marrakech, which was limited by a small sample size (n = 8), the observed median OS was 25.0 months. Four patients achieved a CRc after the first treatment cycle, and the two-year OS was 58%. Although these findings are descriptive and should be interpreted cautiously due to the limited number of patients, they illustrate the feasibility and potential effectiveness of AZA-VEN therapy in a heterogeneous, real-world population, which included de novo AML, patients with prior high-risk myelodysplastic syndrome or myeloproliferative neoplasms, and relapsed/refractory cases. These outcomes reflect careful patient selection, close clinical monitoring, optimized management of hematologic and infectious complications, and individualized VEN dose adjustments in the context of azole antifungal interactions [12,13,23].

Treatment tolerability remains a key challenge. In our cohort, hematologic toxicity was frequent and severe, with grade 3-4 neutropenia, anemia, and thrombocytopenia; all patients experienced febrile neutropenia. Management required dose modifications, close hematologic monitoring, and prophylactic antifungals. Infectious complications remain common, with a high proportion of patients experiencing fever, documented infections, and neutropenia in retrospective cohorts [23,24]. Shorter VEN induction strategies (14 days) have been shown to reduce neutropenia duration, severe infections, and hospitalization without compromising efficacy [24]. Studies evaluating antimicrobial prophylaxis during AZA-VEN induction suggest reduced bacteremia but continued risk of febrile neutropenia and pneumonia [25]. This approach proved particularly useful in elderly patients with comorbidities and limited hospital resources.

Therapeutic failure and disease relapse remain significant obstacles. In our cohort, 50% experienced relapse or progression, reflecting the clinical complexity of elderly AML patients with prior treatment, adverse cytogenetics, and comorbidities. These observations underscore the need for adaptable treatment strategies, longitudinal monitoring, and individualized approaches, potentially including consolidation, maintenance therapies, and allogeneic stem cell transplantation in eligible responders.

Our experience highlights characteristics of the North African setting, including frequent comorbidities, delayed diagnosis, and cytogenetic heterogeneity. Despite limited resources, AZA-VEN proved feasible and effective when intensive monitoring and supportive care were ensured, and dose adjustments were individualized. Incorporation of measurable residual disease monitoring may further optimize patient stratification and inform consolidation or maintenance decisions [16].

Finally, this study demonstrates the real-world feasibility of AZA-VEN in high-risk elderly patients often excluded from international trials. These findings support the need for prospective multicenter studies in North Africa to standardize treatment protocols, assess tolerability and efficacy, and explore novel strategies such as short-course VEN or triplet combinations.

These findings should be interpreted with caution, given the limited sample size and retrospective nature of the study, and are best considered hypothesis-generating rather than definitive.

## Conclusions

Our single-center experience suggests that the combination of VEN and AZA represents a feasible and potentially effective therapeutic option for elderly patients with AML, including those with comorbidities or a prior treatment history. Despite the limited sample size, we observed encouraging remission outcomes and median survival in a real-world setting, broadly consistent with findings from other published real-world series. Hematologic toxicity and infectious complications remain significant challenges, underscoring the need for close monitoring, individualized dose adjustments, and appropriate antimicrobial prophylaxis. Importantly, this study highlights the practical feasibility of implementing AZA-VEN therapy in a North African, resource-limited context, where access to VEN is often restricted by cost. Dose adaptation in combination with azole antifungal therapy may substantially reduce treatment costs, thereby improving accessibility. These exploratory findings support the need for prospective, multicenter studies to confirm efficacy, refine treatment strategies, and further improve survival and quality of life in elderly AML patients.

## References

[1] Yanada M, Naoe T (2012). Acute myeloid leukemia in older adults. Int J Hematol.

[2] Shallis RM, Wang R, Davidoff A, Ma X, Zeidan AM (2019). Epidemiology of acute myeloid leukemia: recent progress and enduring challenges. Blood Rev.

[3] (2026). Cancer Stat Facts: leukemia—acute myeloid leukemia (AML). https://seer.cancer.gov/statfacts/html/amyl.html.

[4] Ferrara F, Schiffer CA (2013). Acute myeloid leukaemia in adults. Lancet.

[5] Cornelissen JJ, Blaise D (2016). Hematopoietic stem cell transplantation for patients with AML in first complete remission. Blood.

[6] Dombret H, Gardin C (2016). An update of current treatments for adult acute myeloid leukemia. Blood.

[7] Kantarjian H, Kadia T, DiNardo C (2021). Acute myeloid leukemia: current progress and future directions. Blood Cancer J.

[8] DiNardo CD, Jonas BA, Pullarkat V (2020). Azacitidine and venetoclax in previously untreated acute myeloid leukemia. N Engl J Med.

[9] Pollyea DA, Amaya M, Strati P, Konopleva MY (2019). Venetoclax for AML: changing the treatment paradigm. Blood Adv.

[10] DiNardo CD, Pratz K, Pullarkat V (2019). Venetoclax combined with decitabine or azacitidine in treatment-naive, elderly patients with acute myeloid leukemia. Blood.

[11] DiNardo CD, Tiong IS, Quaglieri A (2020). Molecular patterns of response and treatment failure after frontline venetoclax combinations in older patients with AML. Blood.

[12] Rausch CR, DiNardo CD, Maiti A (2021). Duration of cytopenias with concomitant venetoclax and azole antifungals in acute myeloid leukemia. Cancer.

[13] Lopez‑Garcia YK, De La Garza F, De la Rosa‑Flores GA (2023). Outpatient low‑dose venetoclax plus azacitidine vs. intensive chemotherapy for newly diagnosed fit patients with acute myeloid leukemia in a limited‑resource setting. Blood.

[14] Ghosh S, Sinha S, Chatterjee S (2024). Outcomes of azacitidine-venetoclax therapy in upfront and relapsed/refractory AML: real-world experience from India. Clin Lymphoma Myeloma Leuk.

[15] Döhner H, Wei AH, Appelbaum FR (2022). Diagnosis and management of AML in adults: 2022 recommendations from an international expert panel on behalf of the ELN. Blood.

[16] Yu H, Wang C, Lei Y (2024). Single-institution experience of venetoclax combined with azacitidine in newly diagnosed acute myeloid leukemia patients. Int Immunopharmacol.

[17] Othman J, Lam HP, Leong S (2024). Real-world outcomes of newly diagnosed AML treated with venetoclax and azacitidine or low-dose cytarabine in the UK NHS. Blood Neoplasia.

[18] Wolach O, Levi I, Canaani J (2020). First results from a nationwide prospective non-interventional study of venetoclax-based 1st line therapies in patients with AML - Revive Study. Blood.

[19] Wolach O, Levi I, Lavie D (2025). Real world prospective observational multicenter trial of venetoclax-based therapy for patients with AML - Revive Study. Blood Adv.

[20] de la Puerta PR, Villegas Da Ros C, Lancharro A (2026). Real-world use of venetoclax + azacitidine in elderly (≥70 years) chemotherapy-ineligible untreated AML patients: a second level hospitals experience. EHA.

[21] Costa A, Baptista RLR, Guaraná M (2026). Clinical characteristics and outcome of patients receiving venetoclax-based regimens in Brazil: a real-world study. Hematol Transfus Cell Ther.

[22] Rattanathammethee T, Chanswangphuwana C, Silpsamrit P (2025). Real-world outcomes of azacitidine plus venetoclax in acute myeloid leukemia: a multicenter retrospective cohort study from Thailand. Ther Adv Hematol.

[23] Zhu LX, Chen RR, Wang LL (2022). A real-world study of infectious complications of venetoclax combined with decitabine or azacitidine in adult acute myeloid leukemia. Support Care Cancer.

[24] Yu Z, Li S, Pei R, Lu Y, Wang Y, Yuan J (2025). Venetoclax combined with azacitidine in elderly acute myeloid leukemia: a retrospective comparison of 14-day vs 28-day dosing regimens. Medicine (Baltimore).

[25] Brandwein J, Liew E, Page D, Wang P (2026). Infectious complications and antibiotic prophylaxis during induction therapy with venetoclax plus azacitidine for previously untreated AML: a real-world experience. Blood.

